# Complete plastome sequence of *Ardisia bullata* G. H. Huang & G. Hao, an endemic species in Hainan

**DOI:** 10.1080/23802359.2020.1778577

**Published:** 2020-06-22

**Authors:** Huan-Xin Wang, Xin-Ru Ke, Rui-Xuan Wang, Hong-Xin Wang, Hong-Liang Zhao

**Affiliations:** a Institute Arts, Sanya University, Sanya, China; b School of Life and Pharmaceutical Sciences, Hainan University, Haikou, China; c Hainan Products Quality Supervision and Testing Institute, Haikou, China

**Keywords:** Ardisia bullata, plastome, phylogeny, genome structure, Primulaceae

## Abstract

*Ardisia bullata* G. H. Huang & G. Hao is a small shrubs of Primulaceae. It is only distributed in Hainan provinces of China. It is a plant medicinal value. There is no study on the genome of *A. bullata* so far. Here, we report and characterize the complete plastid genome sequence of *A. bullata* in an order to provide genomic resources useful for promoting its conservation. The complete chloroplast genome of *A. bullata* is 160,176 bp in length with a typical quadripartite structure, consisting of a large single-copy region (LSC, 89,710 bp), a single-copy region (SSC, 18,357 bp), and a pair of inverted repeats (IRs, 26,054 bp). There are 133 genes annotated, including 83 unique protein-coding genes, eight unique ribosomal RNA genes, and 37 transfer RNA genes. The overall G/C content in the plastome of *A. bullata* is 36.0%. The complete plastome sequence of *A. bullata* will provide a useful resource for the conservation genetics of this species as well as for phylogenetic studies in Apocynaceae.

*Ardisia bullata* G. H. Huang & G. Hao is a shrub of the family Primulaceae. Its distribution range is extremely narrow, only in Hainan provinces of China. It is a plant combines medicinal and dyestuff value (Chen and Pipoly 1996). The chloroplast genome sequence carries rich information for plant molecular systematics and Barcoding. To date, there have been no studies on the genome of *A. bullata.* To provide a rich genetic information and improve *A. bullata* molecular breeding in the future, we report and characterize the complete plastid genome sequence of *A. bullata* (GenBank accession number: MT505713).

In this study, the fresh leaves of *C. sappan* were collected from Diaoluo Mountain in Hainan province (109.91° E, 18.67° N). Voucher specimens (HUTB 187225) were deposited in the Herbarium of the Institute of Tropical Agriculture and Forestry (code of herbarium: HUTB), Hainan University, Haikou, China.

The experiment procedure was as reported in Wang et al. (2019). The total DNA of *A. bullata* was sequenced with second-generation sequencing technology (Illumina HiSeq 2000, San Diego, CA). The chloroplast genome sequence reads were assembled with bioinformatic pipeline including SOAP2 software (Li et al. 2009) and several runs of manual corrections of sequence reads. Genes encoded by this genome were annotated by import the fasta format sequence to the DOGMA (Wyman et al. 2004) and recorrected by manual. The results showed that plastome of *A. bullata* possesses a total length of 160,176 bp with the typical quadripartite structure of angiosperms, containing two Inverted Repeats (IRs) of 26,054 bp, a Large Single-Copy (LSC) region of 89,710 bp, and a Small Single-Copy (SSC) region of 18,357 bp. The plastome contains 133 genes, consisting of 88 unique protein-coding genes, 37 unique tRNA genes, and eight unique rRNA genes. The overall G/C content in the plastome of *A. bullata* is 36.0%, in which the corresponding values of the LSC, SSC, and IR region were 33.40%, 29.90%, and 42.60%, respectively.

We used RAxML (Stamatakis 2006) with 1000 bootstraps under the GTRGAMMAI substitution model to reconstruct a maximum-likelihood (ML) phylogeny of 16 published complete plastomes of Primulaceae, using *Maesa montana* (Primulaceae) as outgroups. According to the phylogenetic topologies, *A. bullata* was closely related to *A*. *solanacea.* Most nodes in the plastome ML trees were strongly supported (Figure 1). The complete plastome sequence of *A. bullata* will provide a useful resource for the conservation genetics of this species as well as for the phylogenetic studies for Primulaceae.

**Figure 1.**
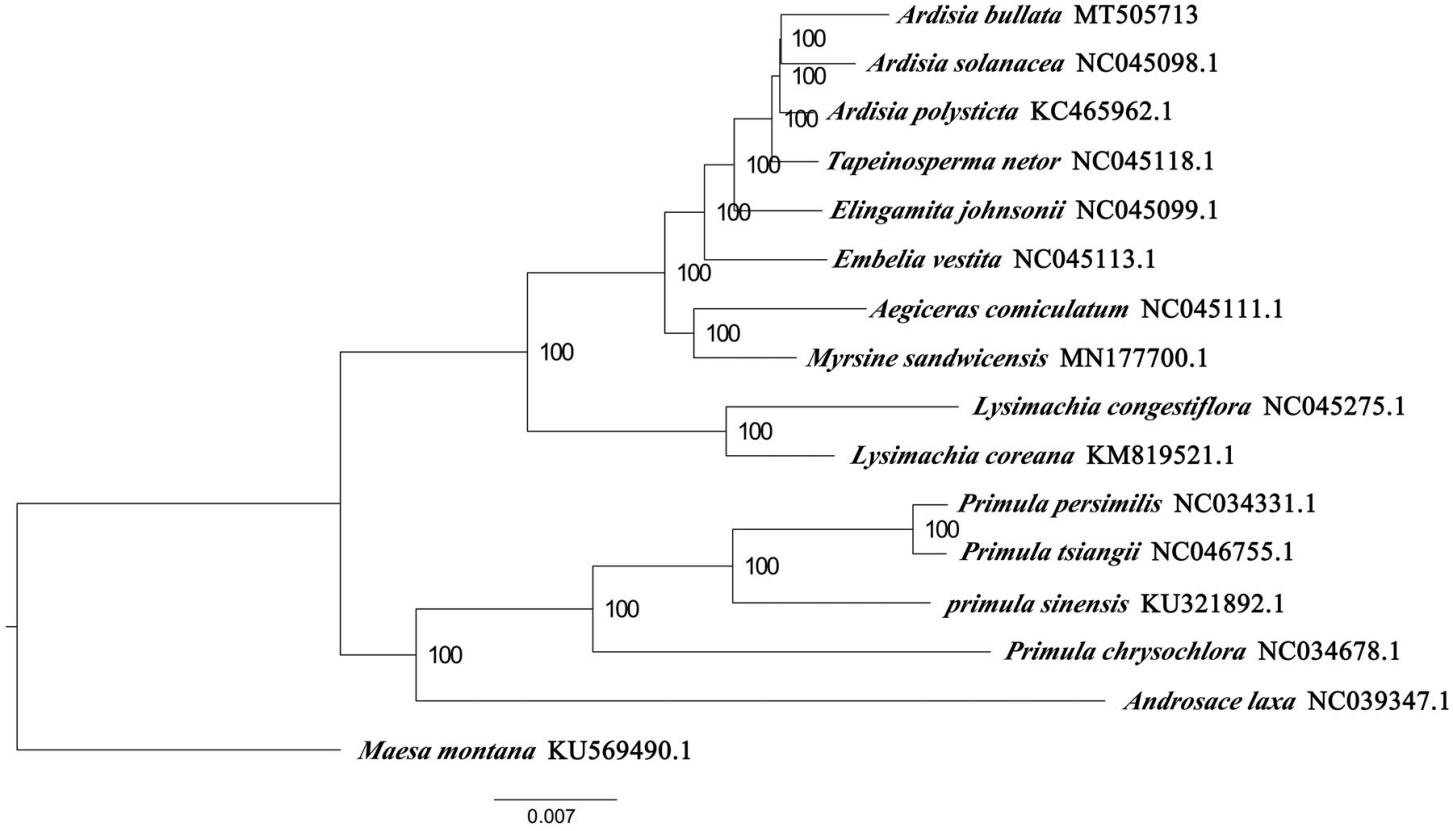
Maximum-likelihood phylogenetic tree based on 16 complete chloroplast genomes. *Ardisia bullata* (this study); *Ardisia solanacea* NC045098.1; *Ardisia polysticta* KC465962; *Tapeinosperma netor* NC045118.1; *Elingamita johnsonii* NC045099.1; *Embelia vestita* NC045113.1; *Aegiceras comiculatum* NC045111.1; *Myrsine sndwicensis* MN177700.1; *Lysimachia congestiflora* NC045275.1; *Lysimachia coreana* KM819521.1; *Primula persimilis* NC034331.1; *Primula tsiangii* NC046755.1; *Primula sinensis* KU321892.1; *Primula chrysochlora* NC034678.1; *Androsace laxa* NC039347.1; outgroup: *Maesa Montana* KU569490.1. The number on each node indicates the bootstrap value.

## Data Availability

The data that support the findings of this study are openly available in GenBank of NCBI at http://www.ncbi.nlm.nih.gov, reference number MT505713.
